# Evaluation of Olfactory Function in Asymptomatic Children With Congenital Cytomegalovirus Infection

**DOI:** 10.7759/cureus.92765

**Published:** 2025-09-20

**Authors:** Chrysanthi-Eleni Loizou, Sofia Karagiannidou, Garyfallia Syridou, Vassiliki Papaevangelou

**Affiliations:** 1 Third Department of Paediatrics, University General Hospital "Attikon", National and Kapodistrian University of Athens, Athens, GRC

**Keywords:** asymptomatic at birth, congenital cmv, congenital cytomegalovirus (cmv), congenital cytomegalovirus infection, olfaction disorders, olfactory dysfunction, postinfectious olfactory dysfunction

## Abstract

Background and aim

Congenital cytomegalovirus (cCMV) is the most prevalent congenital infection worldwide, associated with numerous long-term sequelae, even in infants asymptomatic at birth. However, data are lacking for asymptomatic-at-birth children, who account for the majority of cases. Thus, the aim of the study was to evaluate the olfactory function of asymptomatic cCMV-infected children and compare it to matched healthy controls using a validated odor Identification test.

Methods

A case-control study was conducted. Asymptomatic cCMV-infected children and healthy controls without cCMV history over the age of five were included. Cases and controls were matched at a 1:2 ratio for age and sex. Exclusion criteria included other conditions associated with transient or permanent olfactory dysfunction. A U-Sniff odor identification test (Kids Ident Test "U-Sniff"; Burghart Messtechnik GmbH, Holm, Pinneberg, Germany) was used to evaluate olfaction. Statistical analysis was conducted using IBM SPSS Statistics for Windows, version 25.0 (IBM Corp., Armonk, New York, United States).

Results

Overall, 63 children were included (61.9% females; median age: 7 years). No differences in olfactory function (p=0.657) or olfactory score percentile (p=0.853) were detected between cases and controls. No differences in olfactory function or olfactory score percentile were detected in two sub-analyses, where participants were stratified according to age group (5-8 years old; p=0.545 and p=0.472, respectively, and 9-11 years old; p=1.000 and p=0.088, respectively) and COVID-19 infection history (positive history; p=0.645 and p=0.694, respectively, and negative history (p=not computable and p=0.509, respectively).

Conclusions

Olfactory function of children with cCMV infection who were asymptomatic at birth does not seem to differ from healthy sex-and-age-matched controls.

## Introduction

Congenital cytomegalovirus (cCMV) is the most common viral congenital infection worldwide and a leading cause of non-genetic sensorineural hearing loss (SNHL) and neurodevelopmental impairment (NDI) [1-3]. Symptoms at birth are associated with long-term sequelae; however, 15-20% of children asymptomatic at birth (90%) may also present late-onset sequelae, mainly SNHL [2-4].

Cytomegalovirus (CMV) is a neurotropic virus, with its impact on the developing nervous system being well acknowledged after years of extensive research. Affected children may suffer from SNHL, epilepsy, motor deficits, and mild NDI [2-5]. However, the olfactory function of cCMV-infected children has not been adequately studied. Olfactory bulb malformations have been detected in both a pathology study of infected fetuses [6] and an animal model study [7]. Recently, a cranial MRI of a neonate with symptomatic cCMV infection revealed increased signal intensity in the central portion of the olfactory bulb in the T2 sequence [8], while another study reported reduced olfactory function in cCMV-infected children who are symptomatic at birth, using a house-made odor discrimination test [9].

The aim of this study is to provide data on the olfaction of asymptomatic cCMV-infected children, compared to children without known cCMV infection. To our knowledge, this is the first study attempting to assess the olfactory function of asymptomatic cCMV-infected children using a validated pediatric test.

## Materials and methods

Study design

This was a single-center, prospective, case-control study (1:2) conducted in the Pediatric Infectious Disease Unit of University Hospital “Attikon”, Athens, Greece, between May 2023 and March 2024. The study was approved by the Scientific Council of University General Hospital "Attikon" (reference number: ΕΒ∆233/03-04-2023). The parents of both cases and controls provided written informed consent.

Study population

Cases consisted of asymptomatic cCMV-infected children born before January 2018, prospectively followed up in our center. The age criterion was required since the olfactory test used has been validated for children older than five years [10,11]. cCMV infection was confirmed by a positive urine polymerase chain reaction (PCR) for CMV during the first 21 days of life, while asymptomatic status was defined as lack of cCMV-related clinical symptoms at birth, as per European Congenital Cytomegalovirus Initiative guidelines [4]. Controls were recruited from the Outpatient Pediatric Clinic of our Department between June and October 2023, and they were matched with cases (1:2) for age and sex.

Exclusion criteria for both groups included a history of allergic rhinitis, chronic rhinosinusitis, herpetic meningoencephalitis, traumatic brain injury, and syndromic genetic disorders associated with anosmia. Moreover, both cases and controls were excluded if they presented with an acute respiratory tract infection on the day of the scheduled olfactory test.

Data collection

A data collection form was constructed for the purposes of the study. Demographic data (date of birth, sex) and medical history (including current medication regimen and previous SARS-CoV-2 infection) were noted.

Olfactory assessment

Assessment of olfactory function was conducted using a U-Sniff odor identification test [11] (Kids Ident Test "U-Sniff"; Burghart Messtechnik GmbH, Holm, Pinneberg, Germany). The test consists of 12 cards depicting four different items each and 12 markers, each containing a different mono-odorant. Cards and markers are uniquely matched to each other via a number (1-12) written at their top. The items depicted and odors contained include food, beverages, and plants. Testing was conducted following a standardized process, based on the manufacturer’s instructions. Children were presented with a card and were asked to inform the examiner in case they needed clarification of the items depicted. Then, the respective marker was placed 2 cm below their nostrils for two to three seconds. After its withdrawal, they were asked to point at the item on the card that best described the odor of the marker. The process was repeated 12 times. The sum of correct responses created the olfactory score of the child, which was interpreted as normosmia or reduced olfactory function according to the cut-off provided for the patient’s age; for patients aged 5-8 years, normosmia was defined as a score of 7 or higher, while for patients aged 9-11 years, normosmia was defined as a score of 9 or higher. Additionally, the patient’s olfactory function percentile for age (<10%, 10-50%, and 50-90%, respectively) was determined using age-adjusted charts.

Sample size

Currently, there is no data on the prevalence of olfactory dysfunction in healthy children or among asymptomatic cCMV-infected children. Moreover, the prevalence of olfactory dysfunction in symptomatic cCMV-infected children has been estimated using a house-made test with mono-odorants and binary mixtures that has not been validated for children [9]. Therefore, we used a sample of 21 asymptomatic cCMV children and 42 matched (1:2) controls, based on convenience sampling.

Statistical analysis

Statistical analysis was conducted using IBM SPSS Statistics for Windows version 25.0 (IBM Corp., Armonk, New York, United States). Descriptive statistics are presented as absolute (N) and relative frequencies (%) for categorical variables and as median (25th-75th percentile) for continuous, not-normally distributed variables. Univariate analysis between categorical variables was performed using the Chi-square test of independence (with Fisher’s exact method when one or more expected values were less than 5). The Mann-Whitney non-parametric test was used to make comparisons between categorical variables and continuous, not-normally distributed variables. Significance level was set at α=0.05. Bonferroni correction was applied to adjust for multiple comparisons.

## Results

Overall, 51 cCMV-infected children were eligible for inclusion in our study. We were able to contact 41 parents (six parents could not be contacted since up-to-date contact information was not available, and four did not respond to multiple communication attempts). Of the 41 parents contacted, parents of two children refused participation due to follow-up at another hospital, parents of eight children reported they did not want their child to be tested as they did not present any cCMV-related LTI and they felt testing would be redundant, and 10 children could not be tested because of social reasons (residence outside of Athens and serious family problems unrelated to cCMV that rendered their visit at our hospital difficult). Thus, a total of 21 asymptomatic-at-birth cCMV-infected children were evaluated, leading to a response rate of 51.2%.

Of the total 21 participants, 61.9% were female, while the participants' median age was seven years (range, 5-11 years). The median gestational age of cCMV-infected children enrolled was 38+1 weeks (range, 37+5, 39+1 weeks). One of the children had a comorbidity unrelated to cCMV (increased levels of lipoprotein A), while none of the children were on any medications at the time of assessment. Six children (28.5%) had cCMV-related long-term sequelae; two had isolated SNHL, three had NDI requiring intervention, while one child presented with both conditions. For those children presenting with NDI requiring intervention, it was deemed by developmental medicine assessments that their ability to complete the olfactory test was not impacted. Additionally, 42 healthy controls were recruited. The characteristics of the study cohort can be found in Table 1.

**Table 1:** Cohort characteristics Data have been presented as n (%) except for continuous not-normally distributed variables (gestational age, age), which are presented as median (25th-75th %). p-values were considered significant if <0.05 and are marked in bold (*chi-square test of independence, **Fisher's exact test, ^Mann-Whitney non-parametric test) SNHL: sensorineural hearing loss; NDI: neurodevelopmental impairment; LTI: long-term impairment; N/A: not applicable

Parameters		Cases (n=21), n (%)	Controls (n=42), n (%)	p-value	Statistical numerical value
Sex	Male	8 (38.1%)	16 (38.1%)	1.000*	<0.0001
	Female	13 (61.9%)	26 (61.9%)		
Gestational Age (weeks)	Median (25^th^-75^th^ %)	38+1 (37+5, 39+1)	Information not available	N/A	N/A
Trimester of infection	1^st^	9 (42.9%)	N/A	N/A	N/A
	2^nd^	6 (28.6%)			
	3^rd^	2 (9.5%)			
	Unknown	4 (19%)			
Age (years)	Median (25^th^-75^th^%)	7 (6-9.5)	7 (6-9.5)	1.000^	441,000
Age group	5-8 years	14 (66.7%)	21 (66.7%)	1.000*	<0.0001
	9-11 years	7 (33.3%)	14 (33.3%)		
Comorbidities	Yes	1 (4.8%)	23 (54.8%)	<0.0001*	14,841
	No	20 (95.2%)	19 (45.2%)		
Medication	Yes	0 (0%)	15 (35.7%)	0.005*	9,844
	No	21 (100%)	27 (64.3%)		
COVID-19 infection history	Yes	18 (85.7%)	33 (78.6%)	0.735**	0.463
	No	3 (14.3%)	9 (21.4%)		
LTI	Yes	6 (28.6%)	N/A	N/A	N/A
	No	15 (71.4%)			
Type of LTI	No LTI	15 (71.4%)	N/A	N/A	N/A
	SNHL	2 (9.5%)			
	NDI	3 (14.3%)			
	SNHL and NDI	1 (4.8%)			

Statistically significant differences between cases and controls were noted in terms of comorbidities and medication regimen, which was expected since controls were recruited from an outpatient pediatric clinic. Reasons for visit of controls on the day of recruitment and associated comorbidities can be found in Table 2. Since no olfactory impairment was associated with these conditions, we considered our sample of healthy controls appropriate. 

**Table 2:** Distribution of study controls by pediatric department and reason of visit on the day of recruitment LDL: low-density lipoprotein

Department	Controls (n=42 ), n (%)	Reasons for visit
Pediatric Endocrinology	22 (52.4%)	Hypothyroidism, early puberty, growth hormone deficiency, diabetes mellitus, congenital adrenal hyperplasia, obesity
General Pediatrics	12 (28.6%)	Routine vaccination, routine blood workup; accompanying sibling that was brought for examination
Pediatric Infectious Disease	4 (9.5%)	Parasitic – helminthic infections, recurrent urinary tract infections
Pediatric Lipid Disorders	3 (7.1%)	Increased LDL, increased triglycerides
Pediatric Nephrology	1 (2.4%)	Ureteral stenosis

**Figure 1:**
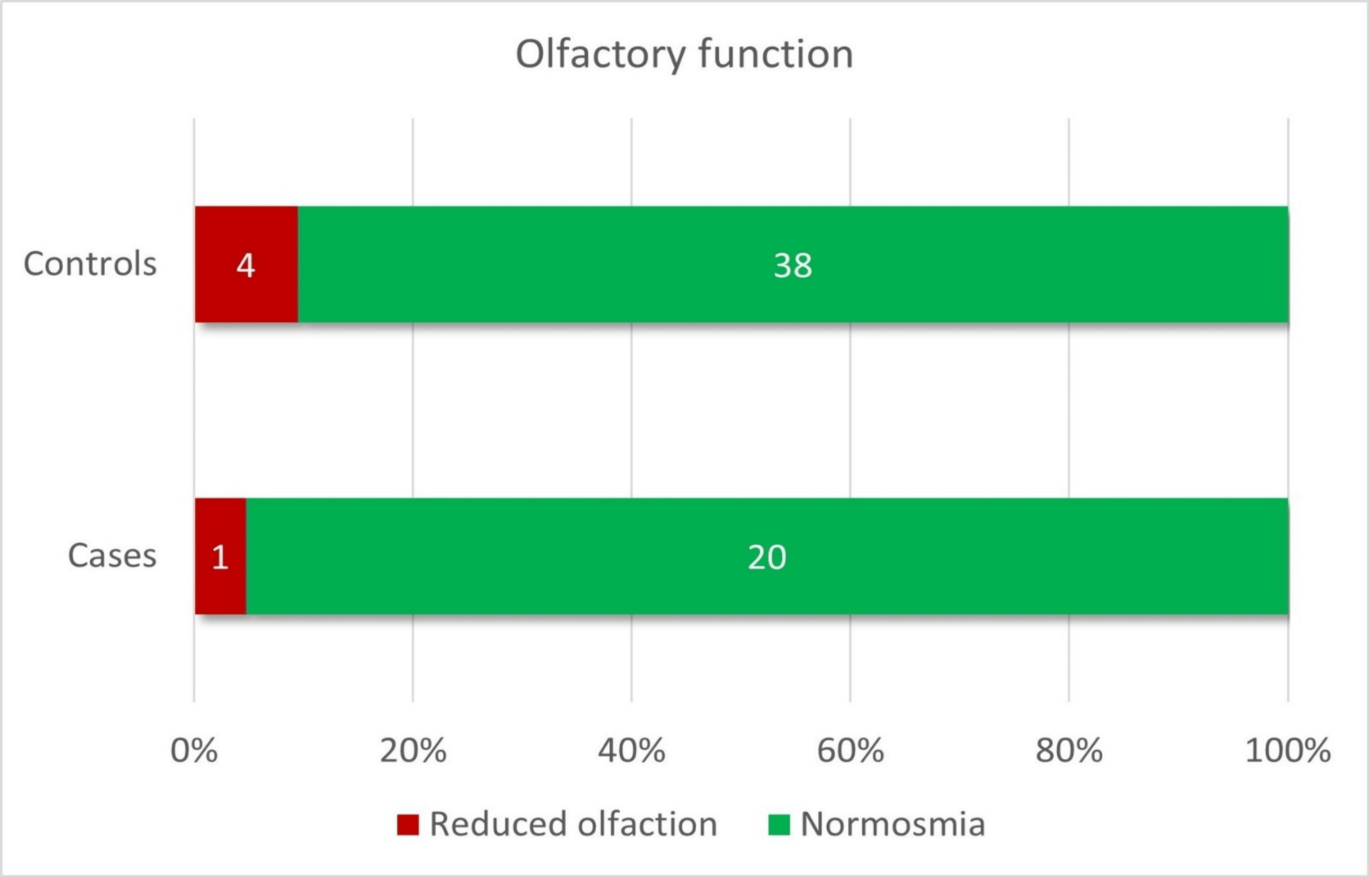
Olfactory function results of the entire cohort Data are represented as absolute (N) frequencies in the bars and relative (%) frequencies in the x-axis. Reduced olfaction is defined as an olfactory score below the 10th percentile for age.

**Figure 2:**
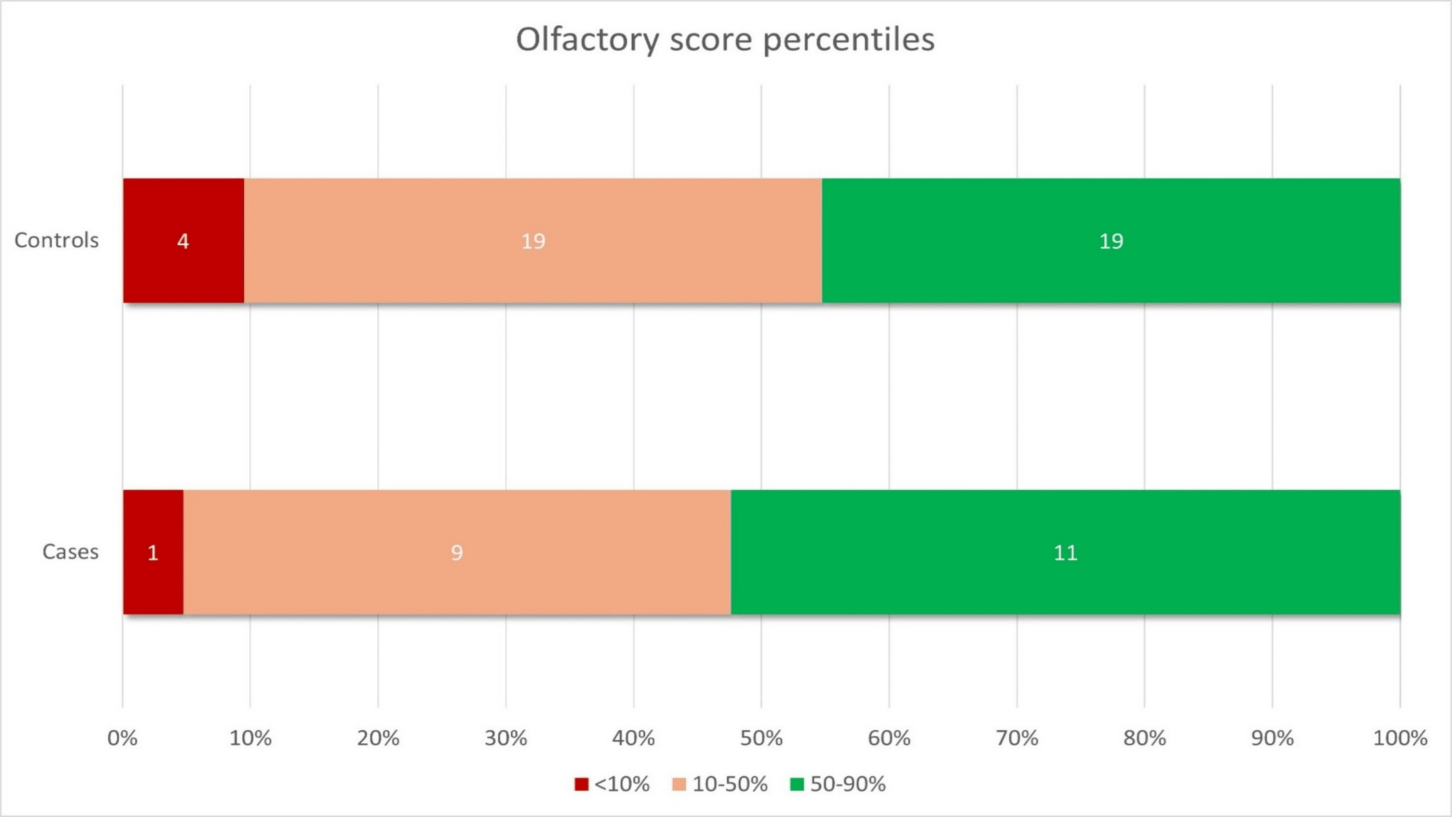
Distribution of olfactory score percentiles in the entire cohort Data are represented as absolute (N) frequencies in the bars and relative (%) frequencies in the x-axis. Reduced olfaction is defined as an olfactory score below the 10th percentile for age.

Parents of all cases and controls reported that their children had a normal sense of smell. According to the 10th percentile threshold of the U-Sniff test for normosmia, one patient in the cCMV group (4.8%) and four patients in the healthy control group (9.5%) presented with a reduced olfactory function (Figure 1). Interestingly, the child in the cCMV group with reduced olfactory function had no long-term sequelae. There were no differences in the olfactory function nor the olfactory score percentiles for age between cases and controls (p=0.657 and p=0.853, respectively) (Figure 1, Figure 2, Table 3).

**Table 3:** Olfactory assessment results Data have been presented as absolute (n) and relative frequencies (%). p-value is considered significant if <0.05 (*Fisher's exact test). N/C: not computable

Parameter		Cases (n=21), n (%)	Controls (n=42), n (%)	p-value	Statistical numerical value
Reported sense of smell	Normal	21 (100%)	42 (100%)	N/C	N/C
	Reduced	0 (0%)	0 (0%)		
Olfactory result	Normosmia	20 (95.2%)	38 (90.5%)	0.657*	0.434
	Reduced olfaction	1 (4.8%)	4 (9.5%)		
Olfactory score percentile	< 10^th^	1 (4.8%)	4 (9.5%)	0.853*	0.500
	10^th^-50^th^	9 (42.9%)	19 (45.2%)		
	50^th^-90^th^	11 (52.4%)	19 (45.2%)		

**Figure 3:**
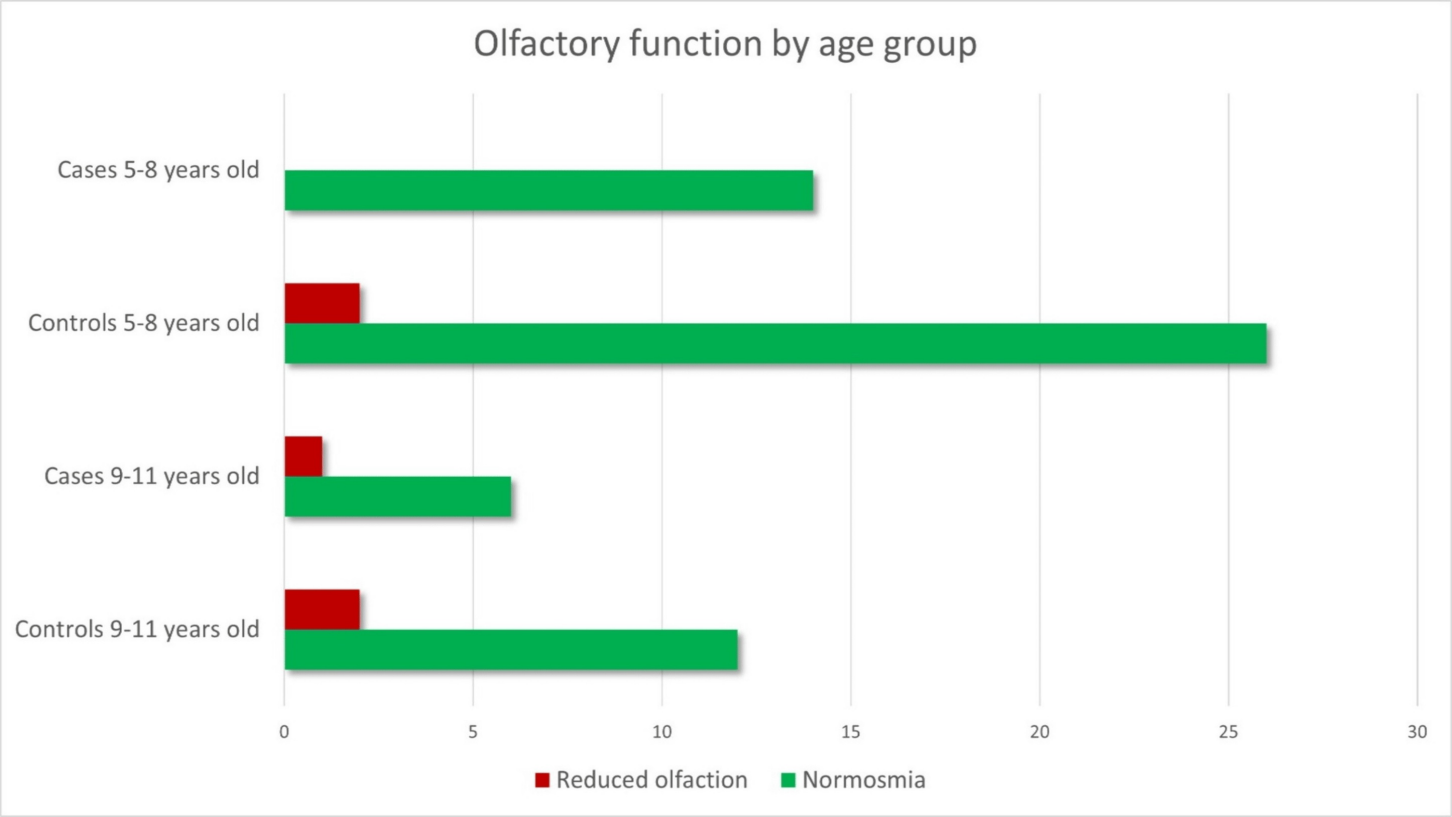
Olfactory function by age group Data are represented as absolute (N) frequencies in the x-axis. Reduced olfaction is defined as an olfactory score below the 10th percentile for age.

**Figure 4:**
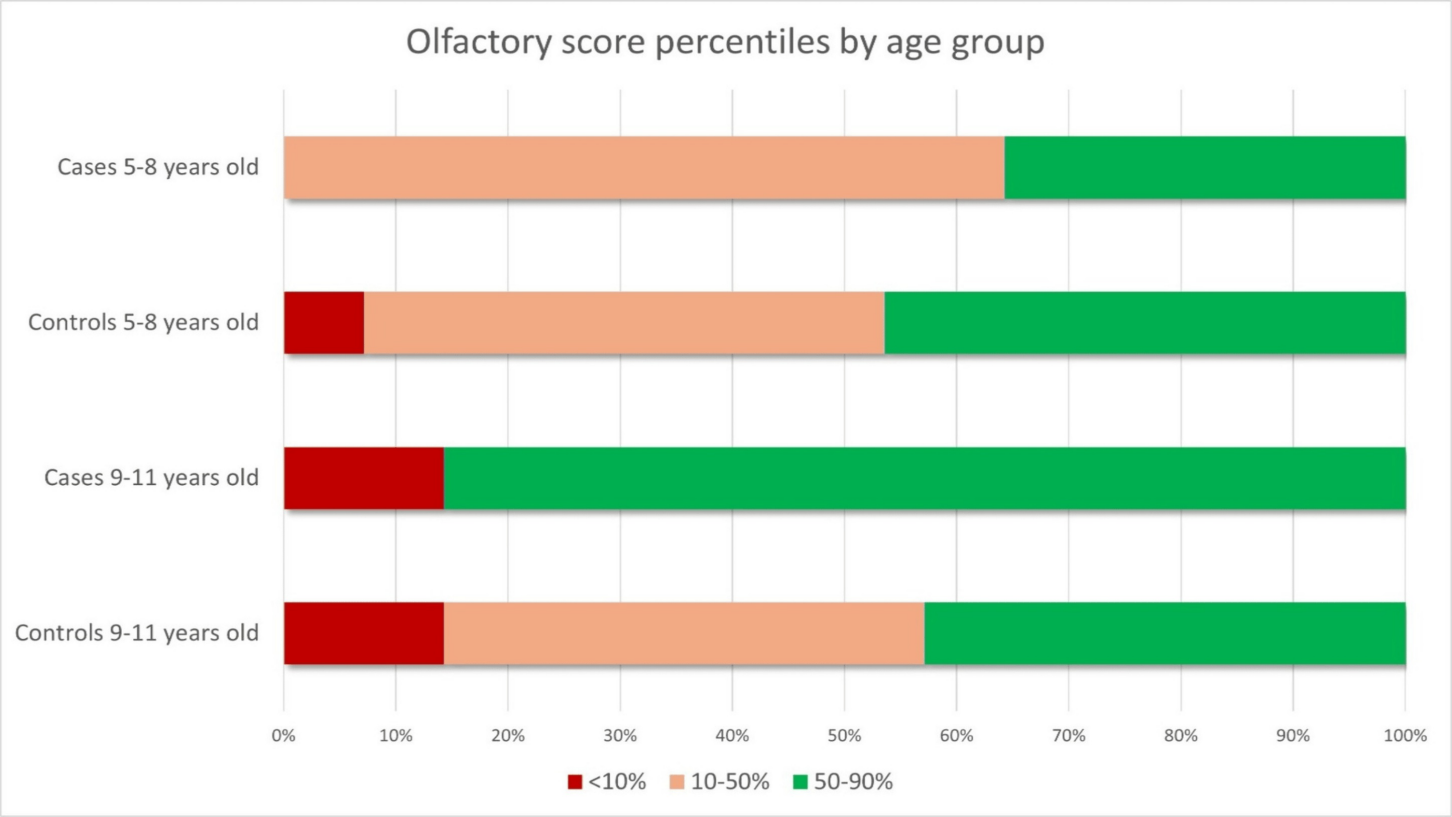
Olfactory score percentiles by age group Data are represented as relative (%) frequencies in the x-axis. Reduced olfaction is defined as an olfactory score below the 10th percentile for age.

No differences were detected in a sub-analysis where participants were divided into two age groups, namely 5-8 and 9-11 years (Figure 3, Figure 4, and Table 4).

**Table 4:** Olfactory assessment results by age group Data has been represented as absolute (N) and relative frequencies (%). p-value is considered significant if <0.05 (*Fisher's exact test). N/C: not computable

Parameter			Cases (n=21), n (%)	Controls (n=42), n (%)	p-value	Statistical numerical value
Reported sense of smell	5-8 years	Normal	14 (100%)	28 (100%)	N/C	N/C
		Reduced	0 (0%)	0 (0%)		
	9-11 years	Normal	7 (100%)	14 (100%)	N/C	N/C
		Reduced	0 (0%)	0 (0%)		
Olfactory result	5-8 years	Normosmia	14 (100%)	26 (92.9%)	0.545*	1,050
		Reduced olfaction	0 (0%)	2 (7.1%)		
	9-11 years	Normosmia	6 (85.7%)	12 (85.7%)	1.000*	<0.0001
		Reduced olfaction	1 (14.3%)	2 (14.3%)		
Olfactory score percentile	5-8 years	< 10^th^	0 (0%)	2 (7.1%)	0.472*	1,431
		10^th^-50^th^	9 (64.3%)	13 (46.4%)		
		50^th^-90^th^	5 (35.7%)	13 (46.4%)		
	9-11 years	< 10^th^	1 (14.3%)	2 (14.3%)	0.088*	4,475
		10^th^-50^th^	0 (0%)	6 (42.9%)		
		50^th^-90^th^	6 (85.7%)	6 (42.9%)		

Finally, we stratified results based on the history of previous SARS-CoV-2 infection to evaluate whether olfaction was affected by COVID-19, confounding our results. Most children, 18 cases (85.7%) and 33 controls (78.6%), reported a known history of SARS-CoV-2 infection. Importantly, all five children with a reduced olfactory function result reported a history of previous SARS-CoV-2 infection. However, the results of our study were not altered when cases and controls were compared based on their reported history of previous SARS-CoV-2 infection (Table 5).

**Table 5:** Stratification of olfactory assessment results by reported SARS-CoV-2 infection history Data has been represented as absolute (N) and relative frequencies (%). p-value is considered significant if <0.05 (*Fisher's exact test). N/C: not computable

Parameter			Cases (n=21), n (%)	Controls (n=42), n (%)	p-value	Statistical numerical value
Reported sense of smell	SARS-CoV-2 infection history	Normal	18 (100%)	33 (100%)	N/C	N/C
		Reduced	0 (0%)	0 (0%)		
	No SARS-CoV-2 infection history	Normal	3 (100%)	9 (100%)	N/C	N/C
		Reduced	0 (0%)	0 (0%)		
Olfactory result	SARS-CoV-2 infection history	Normosmia	17 (94.4%)	29 (87.9%)	0.645*	0.568
		Reduced olfaction	1 (5.6%)	4 (12.1%)		
	No SARS-CoV-2 infection history	Normosmia	3 (100%)	9 (100%)	N/C	N/C
		Reduced olfaction	0 (0%)	0 (0%)		
Olfactory score percentile	SARS-CoV-2 infection history	< 10^th^	1 (5.6%)	4 (12.1%)	0.694*	0.877
		10^th^-50^th^	6 (33.3%)	13 (39.4%)		
		50^th^-90^th^	11 (61.1%)	16 (48.5%)		
	No SARS-CoV-2 infection history	< 10^th^	0 (0%)	0 (0%)	0.509*	1,333
		10^th^-50^th^	3 (100%)	6 (66.7%)		
		50^th^-90^th^	0 (0%)	3 (33.3%)		

## Discussion

cCMV infection is associated with multiple neurologic long-term sequelae, including cerebral palsy, epilepsy, SNHL, and NDI [2-4]. To date, only one study has attempted to evaluate olfactory function in cCMV-infected children [9]. The study used a house-made odor discrimination test to assess olfactory function in symptomatic cCMV children versus controls, demonstrating a significant difference between groups (73.5% of symptomatic cCMV versus 44.1% of controls with a threshold olfactory score <4, p=0.025). However, this test has not been validated for children, the cut-off value used for normosmia was arbitrarily set, and it is not commercially available. Moreover, the study did not include asymptomatic cCMV children.

Assessment of olfactory function is challenging, especially in young children whose attention span is short, linguistic and cognitive skills are not fully developed, and familiarity with odors is limited. Threshold and suprathreshold methods exist for olfactory assessment; suprathreshold methods (odor discrimination and odor identification tests) are used as screening methods, while more specialized tests, including threshold tests, are employed when reduced olfactory function is detected. Odor discrimination tests’ use is limited since no validated tests for children exist. Odor identification tests, which require the child to identify the odorant in a forced-choice manner from pictures or words presented to them, are more frequently used [12]. The U-Sniff Odor Identification Test is the only commercially available test that has been validated for children in 27 countries, including Greece [11,13]. This is the first study to report the use of a validated, standardized odor Identification test to evaluate olfactory function in asymptomatic cCMV-infected children.

Our study was conducted during the post-COVID-19 period, while it has been well established that SARS-CoV-2 can cause olfactory dysfunction [14]. Thus, we suspected that previous COVID-19 infection could be a confounder for olfactory performance. However, the results of this study were not altered when children previously affected by COVID-19 were compared to those with a negative history of a previous SARS-CoV-2 infection. Notably, we did not perform antibody tests to confirm a negative history of previous COVID-19 infection, although this study took place during a period where seropositivity rates among children in Greece were very high [15,16].

Our study has important limitations. First, this is a small study; therefore, differences between cases and controls could be detected in a larger study. Possible recruitment bias cannot be excluded, since a 51.2% response rate was achieved. Also, we cannot exclude that amongst our controls an undiagnosed asymptomatic child with cCMV infection was included, since cCMV screening at birth is not conducted in Greece. Although CMV serology is tested during pregnancy to detect primary maternal infection, the possibility of a non-primary maternal infection cannot be excluded. Additionally, our control group included children with comorbidities, which, even though they have not been associated with an olfactory dysfunction in the literature, their potential influence cannot be completely excluded. Furthermore, we did not perform serology testing to confirm the reported absence of exposure to SARS-CoV-2 infection. Finally, we did not collect data on passive smoking; therefore, such analyses were not conducted.

## Conclusions

Olfactory dysfunction is an important sensory deprivation which has gained popularity in the post-COVID-19 era. In this small, exploratory study, which employed a validated, odor identification test for the first time, no statistically significant differences were detected between asymptomatic cCMV-infected children and healthy controls matched for age and sex. Importantly, this is the first report on the olfactory function of asymptomatic cCMV-infected children. While our data does not support routine olfactory assessment of asymptomatic cCMV-infected children, studies with larger samples are needed in order to confirm these preliminary findings.
